# Phylogeography of freshwater planorbid snails reveals diversification patterns in Eurasian continental islands

**DOI:** 10.1186/s12862-018-1273-3

**Published:** 2018-11-06

**Authors:** Takumi Saito, Takahiro Hirano, Larisa Prozorova, Van Tu Do, Anna Sulikowska-Drozd, Tatiana Sitnikova, Purevdorj Surenkhorloo, Daishi Yamazaki, Yuta Morii, Yuichi Kameda, Hiroshi Fukuda, Satoshi Chiba

**Affiliations:** 1 Graduate School of Life Science, Tohoku University, 41 Kawauchi, Aoba-ku, Sendai, Miyagi 980-0845 Japan; 2 Center for Northeast Asian Studies, Tohoku University, 41 Kawauchi, Aoba-ku, Sendai, Miyagi 980-0845 Japan; 3 Federal Scientific Center of the East Asia Terrestrial Biodiversity, Far Eastern Branch Russian Academy of Sciences, 50 Svetlanskaya Street, Vladivostok, 690950 Russia; 4 Institute of Ecology and Biological Resources, Vietnam Academy of Science and Technology, 18 Hoang Quoc Viet, Cau Giay, Ha Noi, Vietnam; 5 Department of Invertebrate Zoology and Hydrobiology, Faculty of Biology and Environmental Protection, The University of Lodz, Stefana Banacha 12/16, 90-237, Lodz, Poland; 6 Limnological Institute, Siberian Branch Russian Academy of Sciences, 3 Ulan-Batorskaya, Irkutsk, 664033 Russia; 7 WWF Mongolia, Inter Office, Amar Street-4, P.O.Box 20A/115, Ulaanbaatar, 14192 Mongolia; 8 Department of forest Science, Graduate School of Agriculture, Hokkaido University, Kita-9, Nishi-9, Kita-ku, Sapporo, Hokkaido 060-8589 Japan; 9 Center for Molecular Biodiversity Research, National Museum of Nature and Science, 4-1-1 Amakubo, Tsukuba, Ibaraki, 305-0005 Japan; 10 Conservation of Aquatic Biodiversity, Faculty of Agriculture, Okayama University, 1-1-1 Tsushima-naka, Kita-ku, Okayama, 700-8530 Japan

**Keywords:** Biogeography, Immigration, Continental islands, Planorbidae, Freshwater snail, The Japanese archipelago

## Abstract

**Background:**

Islands have traditionally been the centre of evolutionary biological research, but the dynamics of immigration and differentiation at continental islands have not been well studied. Therefore, we focused on the Japanese archipelago, the continental islands located at the eastern end of the Eurasian continent. While the Japanese archipelago is characterised by high biodiversity and rich freshwater habitats, the origin and formation mechanisms of its freshwater organisms are not clear. In order to clarify the history of the planorbid gastropod fauna, we conducted phylogenetic analysis, divergence time estimation, ancestral state reconstruction, and lineage diversity estimations.

**Results:**

Our analyses revealed the formation process of the planorbid fauna in the Japanese archipelago. Most lineages in the Japanese archipelago have closely related lineages on the continent, and the divergence within the Japanese lineages presumably occurred after the late Pliocene. In addition, each lineage is characterised by different phylogeographical patterns, suggesting that immigration routes from the continent to the Japanese archipelago differ among lineages. Furthermore, a regional lineage diversity plot showed that the present diversity in the Japanese archipelago potentially reflects the differentiation of lineages within the islands after the development of the Japanese archipelago.

**Conclusions:**

Although additional taxon sampling and genetic analysis focused on each lineage are needed, our results suggest that immigration from multiple routes just prior to the development of the Japanese archipelago and subsequent diversification within the islands are major causes of the present-day diversity of the Japanese planorbid fauna.

**Electronic supplementary material:**

The online version of this article (10.1186/s12862-018-1273-3) contains supplementary material, which is available to authorized users.

## Background

Islands have long been treated as an excellent model system of evolutionary biology, and critical evolutionary mechanisms that generate species diversity such as adaptive radiation have been revealed by studying islands [1–4]. In considering island biology, immigration is an important event. As in the case of oceanic islands where the biota could not be established without dispersal, dispersal over the ocean or immigration through a land bridge have a large influence on the current biodiversity of continental islands [5–7]. On the other hand, speciation and differentiation within an island also contribute greatly to the diversification of the present fauna [8–14]. The importance of speciation and differentiation within an island have been suggested based on molecular phylogenetic research (e.g. [11, 15–17]). However, most of the studies conducted to date have focused on terrestrial organisms, and the origin of the biodiversity of freshwater organisms on islands it is not well known.

**Fig. 1:**
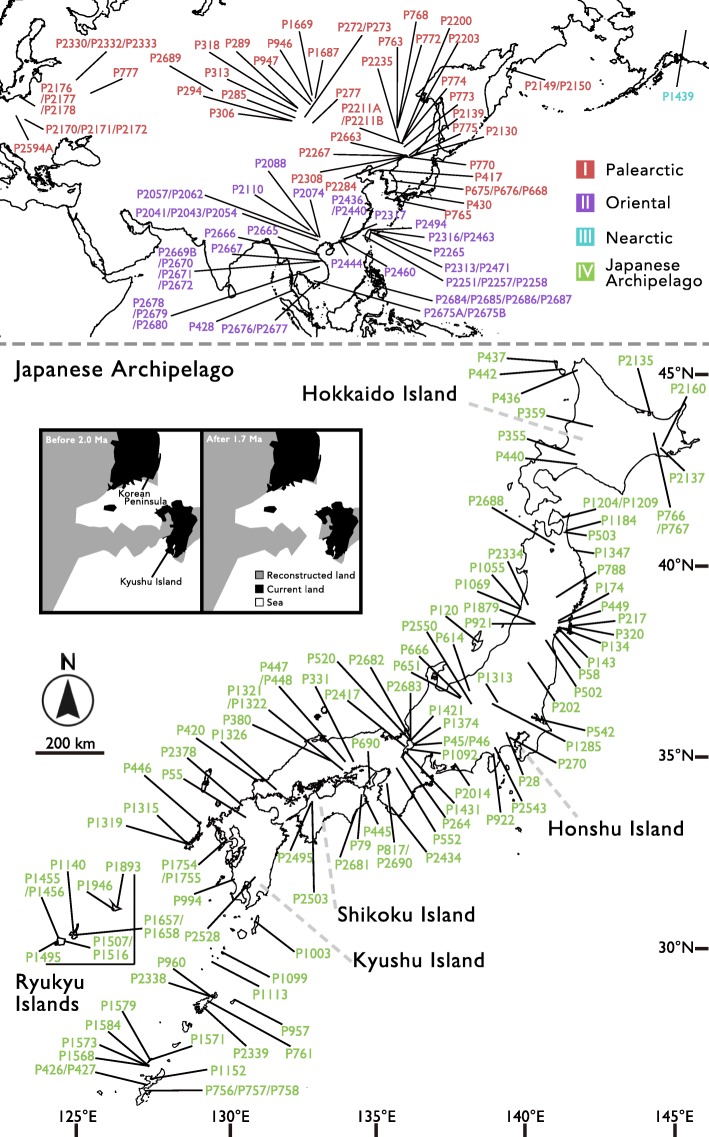
Map of the sample-collection sites. Colours and bars indicate areas where the samples were collected. A paleogeographic map in the lower left box shows geological history of the Japanese archipelago [32]. A sea passage between the continent and archipelago appeared in the southern part of the Japan Sea at 1.7 Ma, and the land bodies of the Japanese archipelago was separated completely from the continent

We accordingly focused on the freshwater fauna of the continental Japanese archipelago (including the Ryukyu Islands). The Japanese archipelago, which includes continental islands located at the eastern end of the Eurasian continent, is considered to be a global hotspot of biodiversity [18]. It is presumed to have formed mainly from land masses that separated from the Eurasian continent about 15 Ma [19–23]. Then, following the uplift of Fossa Magna after 6.0 Ma [24, 25] and the expansion of the Okinawa Trough (2.0–3.0 Ma) [26–28], the cleavage of the southern strait of the Japan Sea occurred 1.7 Ma (Fig. 1) [29–32]. This event rendered the Japanese archipelago a system of isolated islands. There were frequent connections and disconnections with the continent via land bridges due to sea level changes [22, 32–34]. This archipelago and its complex geography are an attractive model system for biogeographical studies, and a lot of research using molecular data has been published in recent years (e.g. [16, 35–43]). However, fewer biogeographical studies have been conducted using freshwater organisms in the Japanese archipelago. Most of these studies have focused on freshwater fishes. These studies have revealed the geographical genetic structure and clarified the formation mechanism of strictly freshwater fish within the Japanese archipelago [28, 44–52]. However, only a few studies have focused on the entire Eastern Eurasian region (e.g. [53, 54]). There have been several biogeographical studies focused on freshwater insects (e.g. [55–58]) and crustaceans (e.g. [59–61]), but information obtained from these investigations is still limited. In the case of freshwater molluscs, one of the most diverse animal groups in the freshwater system, only a few phylogeographical studies have been conducted (e.g. [62–65]). In summary, the biogeography of freshwater organisms in the Japanese archipelago, in particular their origin and comprehensive formation mechanisms, is still not well known.

We accordingly focused on investigating Planorbidae, a group of small freshwater snails. Planorbidae is one of the taxa with the highest species diversity of freshwater molluscs in the Japanese archipelago [66]. Most species in the Japanese archipelago are also found on the Eurasian continent (or closely related species are found) [66–69]. Because freshwater snails have a low active dispersal ability and high passive dispersal ability [70], their geographical genetic structure may have been strongly influenced by a small number of long-range dispersal and diversification events within the islands. Hence, by conducting biogeographical research over the entire Eastern Eurasia region centred on the Japanese archipelago using planorbid snails, we expect to address the origin and diversification mechanisms of freshwater molluscs with few research cases within the continental Japanese archipelago.

## Methods

### Species sampling

We sampled 205 individuals from 163 sites from the Japanese archipelago, Russia, Vietnam, Mongolia, China, Hong Kong, Taiwan, South Korea, Philippines, the United States, Thailand, and Poland for analysis (Additional file 1 and Fig. 1). We used three Hygrophila species as outgroups, referring to the prior researches of higher phylogenetic position within Hygrophila [71, 72]. These samples including outgroups were identified by morphological characteristics using catalogues and lists of freshwater molluscs [66–69, 73]. However, the taxonomy of Planorbidae in East Asia is not fully known, and this investigation is beyond the scope of this research. Therefore, we limited the identification of samples to the genus except for some morphologically clear species. Furthermore, although species that have been hitherto identified as *Gyraulus pulcher* can be clearly distinguished based on their shell morphology [74, 75], the type specimens have some taxonomical problems [Saito T and Fukuda H, unpublished observations]. Hence, *Gyraulus pulcher* was provisionally treated as *Gyraulus* “*pulcher*”. Other *Gyraulus* species, except *G. albus, G. biwaensis* and *G. parvus* were not indentified here. For another Palearctic (mainly Russian) planorbids we used names of genus-group taxa treated by Starobogatov et al. [67]. However, we treated some subgenus instead of genus, because Starobogatov et al. [67] treated *Choanomphalus* as a genus including a number of taxa formerly treated as genera, but in contrast, recent Planorbidae molecular phylogenies [76, 77] showed that some subgenera were not closely related to other subgenenera. These are such genera as *Vitreoplanorbis* and *Pseudogyraulus* first described as subgenera of the genus *Choanomphalus*. In any case, all six genera (including at least 9 species) that were recorded in Japan as native were collected from the Japanese archipelago and these genera were also collected from continental Asia except for *Camptoceras* spp. from South and Southeast Asia. The summarized information of taxon sampling is shown in Table 1. The examined samples were deposited in Tohoku University Museum and Okayama University. Detailed information about the samples is listed in Additional file 1 and in Fig. 1.

**Table 1:** Summarized information of taxon sampling in this study. In addition to these genera and species, we collected 10 related genera (at least including 10 species) and outgroups species

Genus	Nos. of species in Japan	Sampled nos. of species in this study from Japan	Reference	Sampled nos. of species in this study from Japan
*Camptoceras*	1	1	Habe (1990) [107]	Not sampled
*Culmenella*	1	1	Habe (1990) [107]	Russia
*Ferrissia*	1 or 2	1 or 2	Saito et al. (2018) [108]	Russia, Taiwan, Hong Kong, Vietnam
*Gyraulus*	Unclear (At least 4)	4 or more (Geographically covered sampling)	Mori (1938) [109], Habe (1990) [107]	All 11 countries and regions.
*Helicorbis*	1	1	Habe (1990) [107]	Russia, Mongolia, South Korea, Taiwan, Hong Kong
*Polypylis*	1 or 2	1 or 2	Masuda and Uchiyama (2004) [66]	Russia, South Korea, Taiwan, Hong Kong, Vietnam
Total: 6 genera (only native)	Total: at least 9 species (only native)	Total: 6 genera and at least 9 species		

### Molecular methods

Total DNA was isolated from individual gastropods using Nucleospin tissue (TaKaRa, Shiga Pref., Japan) according to the manufacturer’s instructions. To conduct the phylogenetic analyses of Planorbidae, we sequenced fragments of the mitochondrial cytochrome c oxidase subunit 1 (CO1), the mitochondrial large ribosomal subunit (16S) and the nuclear Histone 3 (H3). The conditions of the polymerase chain reaction (PCR) and primers used are listed in Table 2. The PCR products were purified using Exo-SAP-IT (Amersham Biosciences, Little Chalfont, Buckinghamshire, UK). Sequencing was performed using a BigDye™ Terminator Cycle Sequencing Ready Reaction Kit (Applied Biosystems, Foster City, CA, USA) and electrophoresed using an ABI 3130xl sequencer (Applied Biosystems, Carlsbad, CA, USA). The obtained CO1, 16S and H3 sequences have been deposited in the DDBJ/EMBL/GenBank database (Additional file 1).

**Table 2:** Information on primers and PCR conditions used in this study

Primer	Direction	Sequence5’-3’	PCR condition	Reference
CO1				
LCO1490	Forward	GGTCAACAATCATAAAGATATTGG	94 °C 4 min, (94 °C 30 s, 48 °C 30 s, 72 °C 90 s) × 34, 72 °C 2 min	Folmer et al. [110], PCR condition was slightly modified.
HCO2198	Reverse	TAAACTTCAGGGTGACCAAAAAATCA		
16S				
16Sar-L	Forward	CGCCTGTTTATCAAAAACAT	94 °C 4 min, (94 °C 30 s, 40 °C 30 s, 72 °C 60 s) × 34, 72 °C 5 min	Palmubi et al. [111], PCR condition was slightly modified.
16Sbr-H	Reverse	CCGGTCTGAACTCAGATCACGT		
H3				
H3PulF	Forward	GGAGGCAAGGCCCCACGTAARCA	94 °C 3 min, (94 °C 15 s, 57 °C 30 s, 72 °C 40 s) × 40, 72 °C 1 min	Uit de Weerd and Gittenberger [112].
H3PulR	Reverse	TTGGCGTGGATGGCGCACARG		

### Phylogenetic analyses

There was no gap in the alignment of CO1 and H3 except for a 15-bp insertion (this region was removed) and a 9-bp deletion of a few planorbid species in the CO1 sequences. These sequences were aligned with MUSCLE v3.8 [78]. To eliminate uncertainty of the 16S alignment, trimAl 1.2 [79] was used to select regions of the aligned sequences for analysis (Additional file 2). The phylogenetic trees were obtained using Bayesian inference (BI), maximum likelihood (ML), and neighbour-joining (NJ) methods. Prior to the BI and ML analyses, we used the program Kakusan4–4.0.2011.05.28 [80] to select the appropriate models of sequence evolution (Table 3). Based on these models, ML analysis was performed using RaxML [81] and Phylogears2, v2.2.2012.02.13 [82] software referring to recommended in the manual. For the ML analyses, we assessed nodal support by performing bootstrap analyses with 1000 replications. The BI analysis was performed using MrBayes v3.1.2 [83], with two simultaneous runs. Each run consisted of four simultaneous chains for eight million generations and sampling of trees every 100 generations. We discarded the first 8001 trees as burn-in after examining convergence and effective sample size (ESS) using Tracer v. 1.6 [84]; the remaining samples were used to estimate phylogeny. Then, the topologies of each single-locus tree (Additional files 3, 4, 5) were examined. There were no major inconsistencies among the analysed sequences in supported tree topology of three loci, with the proviso that the H3 tree had low resolution. Accordingly, phylogenies using the combined locus were estimated. The same protocols as in single-locus analysis were used in the combined-locus analysis. The selected model is also listed in Table 3. The ML analysis was performed with 1000 replications of the bootstrap analyses. The two simultaneous runs in the BI analysis consisted of four simultaneous chains for 18,000,000 generations and sampling of trees every 1000 generations. We discarded the first 3101 trees as burn-in after checking by Tracer v. 1.6, and the remaining samples were used to estimate phylogeny.

**Table 3:** Information of models of sequence evolution for maximum likelihood and Bayesian analysis

Alignment	Model of sequencing evolution: BI	Model of sequencing evolution: ML
For single tree		
CO1 (Codon Position 1/2/3)	GTR + Γ/ F81 + Γ/ GTR + Γ	GTR + Γ / GTR + Γ / GTR + Γ
16S	GTR + Γ	GTR + Γ
H3 (Codon Position 1/2/3)	SYM + Γ/SYM + Γ/SYM + Γ	GTR + Γ / GTR + Γ/ GTR + Γ
For combined tree		
CO1 (Codon Position 1/2/3)	GTR + Γ/ F81 + Γ/ GTR + Γ	GTR + Γ / GTR + Γ/ GTR + Γ
16S	GTR + Γ	GTR + Γ
H3 (Codon Position 1/2/3)	SYM + Γ + I/SYM + Γ/SYM + Γ	GTR + Γ/ GTR + Γ/ GTR + Γ

### Divergence time estimation and ancestral state reconstruction

We estimated divergence time and conducted ancestral state reconstruction simultaneously using BEAST2 v. 2.4.4 [85] with same dataset with the following settings: tree prior = Yule process; ngen = 20,000,000; samplefreq = 1000; clock models = uncorrelated lognormal relaxed clock. Substitution models of each partition were set as follows: CO1 = GTR + Γ + I, 16S = GTR + Γ, H3 = GTR + Γ. These models were selected using Kakusan4–4.0.2011.05.28 [80] from available evolutionary models in BEAST2 v. 2.4.4 [85]. In addition, the CO1 model was chosen from models in which the molecular clock rate was considered by Wilke, Schultheiß & Albrecht [86]. This molecular clock rate is an average clock rate among the lineages of Protostomia. This rate is also very close to the reliable clock rate obtained by fossil-based calibration in freshwater molluscus [87]. A molecular clock rate (uniform prior) ranging from 0.0125–0.0206 (substitutions per site and My) was proposed for the COI gene for different Protostomia groups referring to the substitution models GTR + Γ + I (see [86]). The geographical region for the ancestral state reconstruction was determined as follows: Palearctic = I, Oriental = II, Nearctic = III, Japanese Archipelago = IV. In our analysis, six BEAST2 runs were conducted with same settings and combined using LogCombiner v. 2.4.4 (BEAST package). Finally, maximum clade credibility files were annotated in TreeAnnotator v. 2.4.4 (BEAST package; burn-in = 10%) summarizing the entire posterior distribution and ancestral state probability including a total of 108,005 trees after log and tree files were checked with Tracer v. 1.6 [84].

### Estimating lineage diversity at internal nodes

To measure lineage diversity at internal nodes in the Japanese archipelago, we conducted three steps of analyses developed by Mahler et al. [88]. In step 1, we estimated the geographical location probabilities of each node in the tree using a Bayesian ancestral state reconstruction that we analysed using BEAST2 v. 2.4.4 [85] (see Section “Divergence time estimation and ancestral state reconstruction”). In step 2, we summed the location probabilities estimated for each region at all earlier nodes, obtaining lineage richness estimates for each region at each time. In step 3, we calculated the product by element of the vector of regional lineage richness at the focal time (from step 2) and the vector of location probabilities from the focal node (from step 1) to obtain the lineage diversity at the focal node. This final sum is the weighted mean of the estimated lineage diversities at each region at each time of our focal node. In addition, to clarify the mechanism of lineage diversification in the Japanese archipelago, we identified branches that occurred within the Japanese archipelago. We did not process nodes with location probabilities less than 0.70.

## Results

### Phylogenetic relationships

**Fig. 2:**
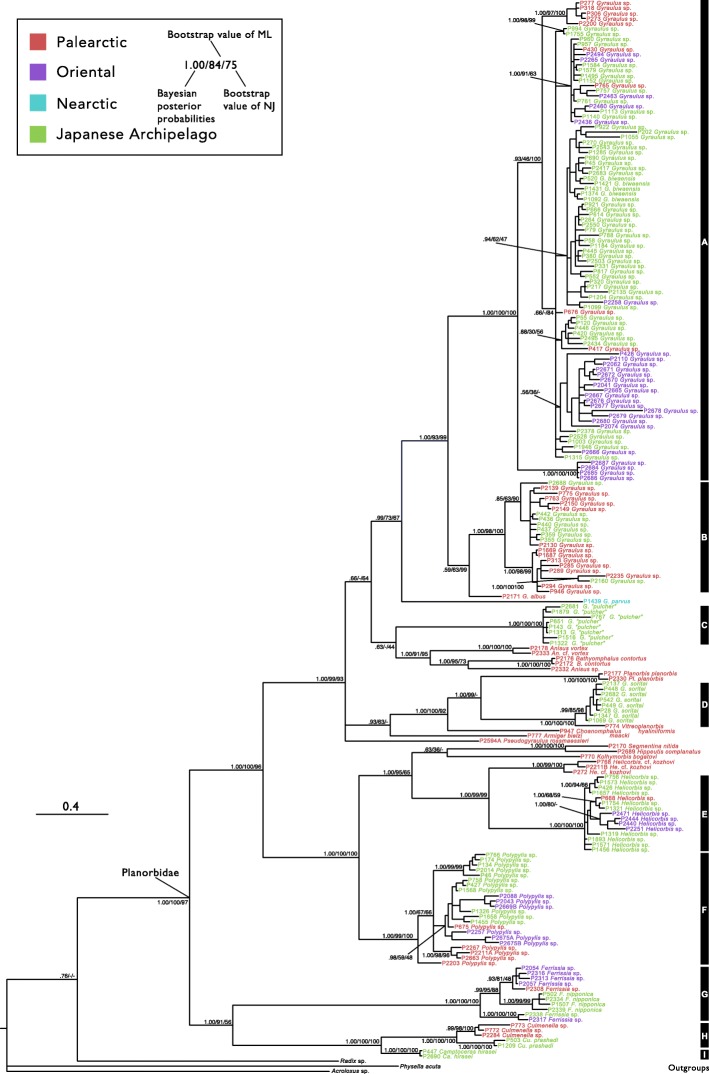
The Bayesian phylogenetic tree inferred from a combined dataset of mtDNA and nDNA sequences (CO1, 16S, and H3; 1375 bp). *Radix* sp., *Physella acuta*, and *Acroloxus* sp. are the outgroups chosen for the tree root. Each number and colour at the terminal branch of the tree indicates the sample number, species name and collected region (Fig. 1 and Additional file 1). Numbers at the branch nodes represent BPP, MLBV, and NJ. On the right side, the vertical bars indicate nominal clades

For the molecular phylogenetic analyses, the ESS values visualized in Tracer v. 1.6 were higher than 200. The inferred Bayesian phylogenetic relationships are shown in Fig. 2. All three estimated trees (BI, ML, and NJ) resulted in nearly identical topologies. The planorbid species from the Japanese archipelago included nine major clades, which we refer to as “A” to “I.” The monophyly of the nine clades was almost fully supported by all three methods. Seven of those nine clades consisted of Japanese samples and Eurasian samples, and the remaining two clades (C and I) consisted only of samples from the Japanese archipelago.

Clades A and B were composed of only *Gyraulus* spp. and these two clades were sister groups. The samples in clade A were collected from Palearctic, Oriental, and the Japanese archipelago. Although there were some supported monophyletic subclades, which tend to be united with samples in nearby regions, the phylogenetic relationships of inner clade A were not sufficiently resolved because many branches had low support values. The samples in clade B were collected from Palearctic regions and the Japanese archipelago. Clade B was subdivided into two subclades. One subclade was composed of mainly Palearctic samples (with the exception of one sample from the Japanese archipelago), and it was strongly supported. Another subclade consisted of both samples from the Japanese archipelago and the Palearctic region, but it was not sufficiently supported by BI (Bayesian posterior probabilities (BI) = 0.85). Clade C included only *G.* “*pulcher*” from the Japanese archipelago. This clade was a sister to the supported clade of European *Anisus* and *Bathyomphalus*, but it not sufficiently supported and its sister group was unclear. Clade D consisted of monophyletic *G*. *soritai* from the Japanese archipelago and *Vitreoplanorbis hyaliniiformis* from Palearctic. This clade was a sister to the monophyletic *Planorbis planorbis*. This monophyly was supported by the BI and ML methods (BI = 1.00, ML bootstrap value (BV) = 75). Clade E was composed of *Helicorbis* spp. from the Japanese archipelago and Palearctic and Oriental regions, and clade F was composed of *Polypylis* spp. from the Japanese archipelago and Palearctic and Oriental regions. These clades included some regionally supported subclades. Clade G consisted of *Ferrissia* spp. from the Japanese archipelago and Palearctic and Oriental regions and *F. nipponica* from the Japanese archipelago. Continental *Ferrissia* sp., Japanese *F*. *nipponica,* and *Ferrissia* sp. from Oriental regions and the Japanese archipelago created a well-supported monophyly. In addition, Continental *Ferrissia* sp. and Japanese *F*. *nipponica* were also a monophyly. Clade H consisted of Continental *Culmenella* sp. and Japanese *Cu*. *prashadi*, which were a strongly supported monophyly. Finally, *Camptoceras hirasei* was a strongly supported monophyly (clade I), and these two lineages exhibited a sister relationship.

### Divergence time estimation and ancestral state reconstruction

**Fig. 3:**
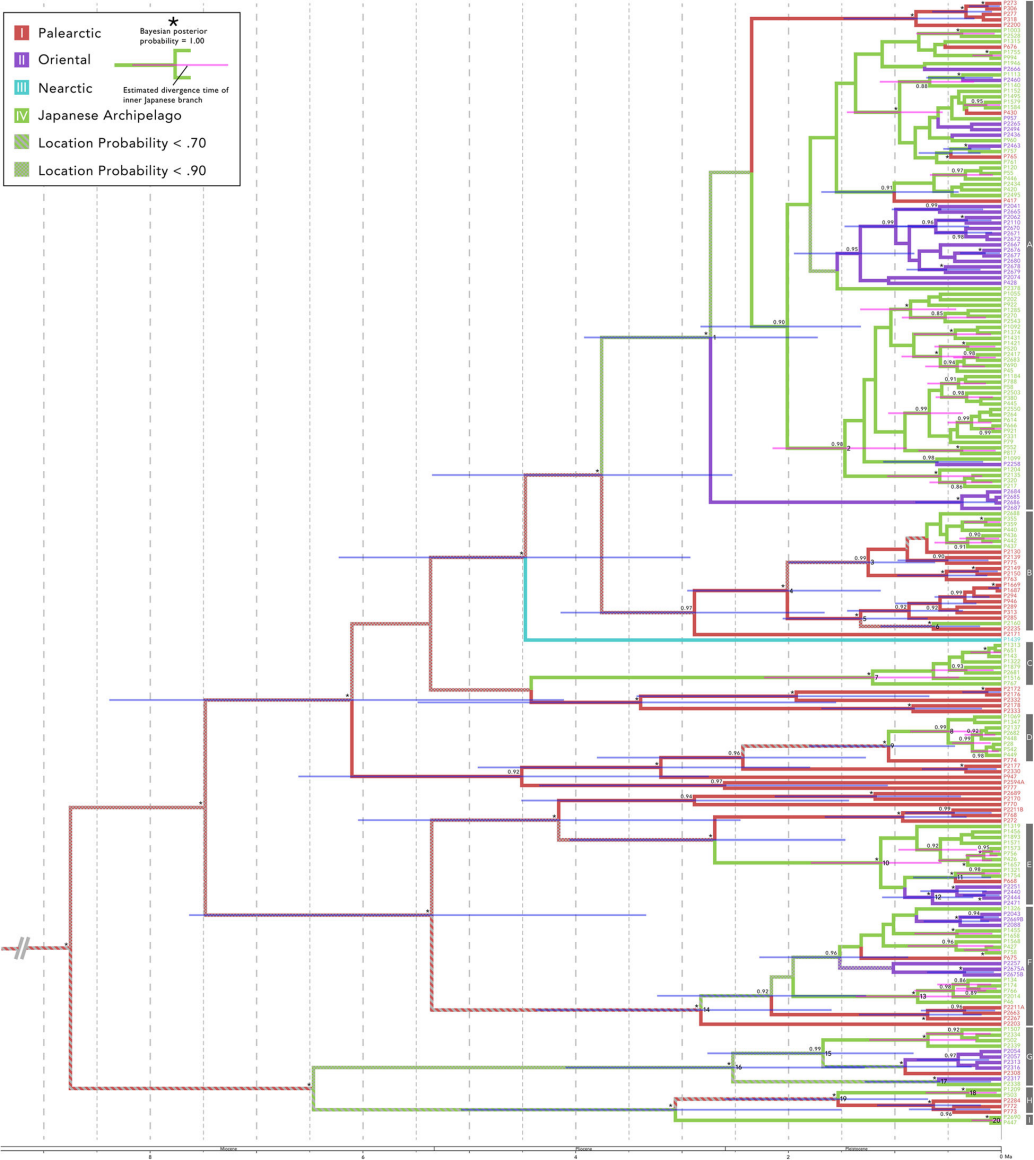
Maximum clade credibility tree generated by the BEAST2 analysis from the mtDNA and nDNA sequences (CO1, 16S, and H3; 1375 bp). The outgroups are not shown. On the right side, sample numbers and nominal clades are listed. Colour indicates the region of the collected samples or an estimation of region according to ancestral state reconstruction. The branches with a low location probability (< 0.70) and a high location probability (0.90) are shown using a striped pattern and a grid pattern, respectively. Node bars indicate 95% CI of the divergence time, and pink node bars indicate branches within the Japanese archipelago. The numbers or marks on the left side of each node indicate BPP. The BPP and node bars are only shown for the relatively supported (BPP > 0.90) nodes. The numbers on the right side of the nodes are the nominal clade number. In the lower part of the graph is the geologic time scale

For the molecular clock analyses, the ESS values visualized in Tracer v. 1.6 were considerably higher than 200. The inferred Bayesian phylogenetic relationships using BEAST2 appear in Fig. 3 and Table 4. The tree topology was nearly consistent with that obtained in the MrBayes and RaxML analyses (Fig. 2). In particular, the major nine clades (clades A through I) were enough supported (BPP > = 0.97) again, and the samples included in these clades were completely consistent with other phylogeny.

**Table 4:** Detailed results of divergence time estimation and ancestral state reconstruction. Significant figure was decided to be three digits except for BPP. See also Additional file 6

Node No.	Divergence Time	BPP	Estimated regional state at each node				Lineage Diversity
	Mean (95% CI; Lower, Upper)		I	II	III	IV	
1	2.73 (1.73, 3.92)	1.00	0.259	0.0229	0	0.718	5.84
2	1.47 (0.889, 2.15)	0.98	0.00110	0.00110	0	0.998	13.2
3	1.32 (0.692, 2.06)	1.00	0.970	0	0	0.0302	19.8
4	2.01 (1.13, 2.95)	1.00	0.931	0.000100	0	0.0680	16.5
5	1.25 (0.623, 2.00)	0.99	0.869	0.000300	0.000200	0.130	20.4
6	0.639 (0.200, 1.13)	1.00	0.891	0	0	0.109	32.3
7	1.21 (0.398, 2.23)	0.99	0.0448	0.00680	0	0.949	17.5
8	0.500 (0.195, 0.857)	0.99	0.0186	0.000100	0	0.981	58.0
9	1.06 (0.435, 1.81)	1.00	0.687	0.00100	0	0.312	22.6
10	1.13 (0.561, 1.79)	1.00	0.0160	0.0447	0	0.939	18.6
11	0.431 (0.0971, 0.829)	1.00	0.0471	0.00140	0	0.952	65.9
12	0.647 (0.264, 1.12)	1.00	0.000300	0.931	0	0.0691	9.63
13	0.786 (0.293, 1.37)	1.00	0.0192	0.00130	0	0.980	34.3
14	2.82 (1.60, 4.38)	1.00	0.622	0.00560	0.000100	0.372	9.12
15	1.68 (0.825, 2.76)	0.99	0.0389	0.131	0.000100	0.830	8.50
16	2.52 (1.28, 4.10)	1.00	0.0777	0.167	0.000300	0.755	4.44
17	0.590 (0.0970, 1.28)	1.00	0.00480	0.316	0	0.680	36.3
18	0.319 (0.0443, 0.711)	1.00	0.0113	0.000400	0	0.988	89.7
19	1.53 (0.690, 2.60)	1.00	0.526	0.00570	0	0.468	15.7
20	0.0977 (0.00180, 0.272)	1.00	0.000800	0.000100	0	0.999	121

In most cases, divergence first occurred between the lineages of the Asian continent and those of the Japanese Archipelago, and then divergence occurred within the lineages of the Japanese archipelago. The mean divergence times of the lineages of Asian continent and those of the Japanese Archipelago were early to middle Pleistocene (e.g. nodes 1, 9, 14 and 19). In contrast, divergence of the lineages within the Japanese Archipelago occurred after middle Pleistocene (e.g. nodes 8, 13, 18 and 20). These results of the dominant nodes are listed in Table 4. Other all results are listed in Additional file 6.

### Lineage diversity

**Fig. 4:**
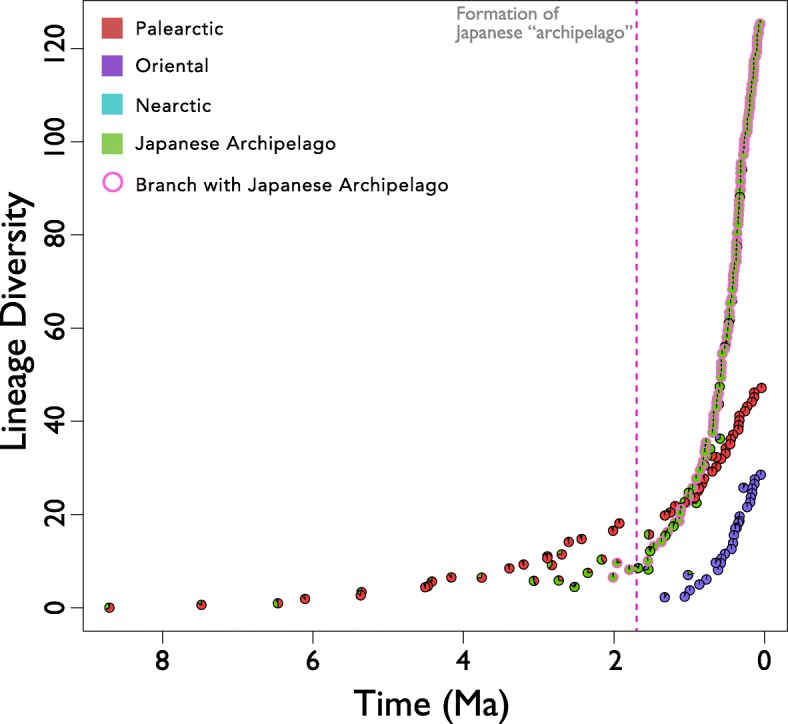
The lineage diversity plot using the method of Mahler [88]. The vertical axis indicates lineage diversity, and the horizontal axis indicates time. The pie chart shows the ancestral regional state in each node in ancestral state reconstruction analysis (Fig. 3). The colour indicates region. The circles with pink outline showed nodes that have a high probability Japanese ancestral state. The dotted pink line indicates the time that the Japanese archipelago formed as an archipelago by the cleavage of the south strait of the Japan Sea (1.7 Ma) [29–32]

The estimated lineage diversity in the Japanese archipelago seems to have manifested around 3.0 Ma and diversified after 1.7 Ma (Fig. 4).

## Discussion

Our phylogeny clearly showed that planorbid snails in the Japanese archipelago do not have a single origin because samples from the Japanese archipelago constitute several monophyletic groups with continental samples at the nominal genus or species level. These clades have different biogeographical patterns, and they appear to be roughly divided into four types: northern clades (Figs. 2 and 3; clades B, D, and H), southern clades (Figs. 2 and 3; clades E and G), widely distributed clades (Figs. 2 and 3; clades A and F), and the endemic Japanese archipelago clades (Figs. 2 and 3; clades C and I). The northern clades consist of samples from the Japanese archipelago and the Palearctic. These samples from the Palearctic were collected in the northern part of the region, and these clades were located mainly in high-latitude areas. In contrast, the southern clade was composed of samples from the Japanese archipelago and Oriental and South Korea. This clade also seems to be distributed in mainly low-latitude areas in eastern Eurasia. On the other hand, widely distributed clades included samples from a wide range of eastern Eurasia. In these clades, regional subclades tend to be formed. Finally, the clades found only in the Japanese archipelago did not have closely related sister group in our analyses. However, *Camptoceras* spp. from the continent were not collected in this research, although this lineage is clearly distinguished by its shell morphology [89]. As a result, clade I is not discussed in this study. On the contrary, our phylogeny indicates that clade C may be an endemic lineage, although further investigation is needed.

These biogeographical patterns may be related to the origins of the planorbid populations in the Japanese archipelago. Japanese populations included northern clades and southern clades. The former originated on the northern Eurasian continent, and the latter originated on the southern Eurasia continent. The geological history of the Japanese archipelago supports this hypothesis. The Japanese archipelago was often connected with the Eurasian continent via a land or ice bridge [22, 32]. The following three conjunctions are known to be major routes with the continent: the south route via the Ryukyu archipelago, the north route via Sakhalin and the Kuril archipelago, and another route via the Korean Peninsula [22]. The Japanese population in the northern clade may have been established via immigration from the northern route, and likewise the Japanese population in the southern clade may have originated from the southern route. Furthermore, perhaps the Japanese population in widely distributed clades may be derived from the Korean Peninsula route. These biogeographical patterns have been noted from fossil records or comparisons of animal fauna between the Japanese archipelago and the continent (e.g. [90–94]) and, more recently, molecular phylogenetic studies of various taxa have provided certain evidence for this possibility (e.g. [28, 39, 41–43, 95]). We estimated the ancestral regional state at each branch that splits into Japanese and continental lineages, but our results were not necessarily clear. The ancestral regional state at the branches of clades B and E was estimated to be region I (Palearctic) and IV (the Japanese archipelago) with a relatively high probability (>.90). In addition, immigration from the Japanese archipelago to the continent was estimated in some branches within some major clades. Although these results appear to fluctuate depending on taxon sampling or regional classification, immigration from the Japanese archipelago to the continent may have occurred. Although immigration from the Japanese archipelago to the continent has often not been taken into consideration in the past, its importance has begun to be pointed out in recent years [28, 96], consistent with our analysis.

We have addressed the question as to when planorbid fauna in the Japanese archipelago were established. Our analysis suggests that the divergence time within major clades except for clade I was around the late Pliocene to the early Pleistocene. In particular, the estimated mean divergence time was concentrated between 2.0 and 2.5 Ma. In these clades, it is not easy to decide which branch emigrated from the continent to the Japanese archipelago. However, our analysis suggests that the foundation of the Japanese population occurred around 2.0–2.5 Ma, and this time scale may result from the development of the Japanese archipelago. The land bodies that formed the Japanese archipelago first separated from the continent 15 Ma [19–23]. During this event, the land bodies of the Japanese archipelago still partially connected with the Asian continent. A sea passage was developed in the southern part of the Japan Sea at 1.7 Ma [29–32], and the land bodies of the Japanese archipelago was separated completely from the continents (Fig. 1). This dramatic geological event occurred 1.7 Ma [29–32]. The planorbid fauna present in the Japanese archipelago today appear to be strongly influenced by the immigrations that occurred before Japan became an “island.” However, the estimated divergence time of a lot of the branches between the Japanese archipelago and the continent (e.g., nodes 6, 11, and 17) post-dates this period. These biogeographical patterns are likely to have been formed via immigration through the several connections from the continent after the Japanese archipelago had been established in its present-day location. Actually, since 1.7 Ma the Japanese archipelago has been sometimes connected with the continent [22, 32–34]. In addition, incidental long-range dispersal of freshwater snails due to birds, wind, ocean current, and desalination of the ocean should also be considered. In particular, dispersal by birds (i.e., snails attached to birds or eaten by birds) has been shown to be possible experimentally [97–100]. In fact, our phylogeny also indicated that gene flow of both regions could occur when the Japanese archipelago and the continent were temporally connected.

Despite the influence from the continent noted above, our results suggest that diversification of the Japanese planorbid lineages within the islands may be the main cause of their present-day diversity (Fig. 4). Although the time in this figure only use the mean estimated divergence time in BEAST2, our results suggest that differentiation within the island may have played a key role in lineage diversification despite the high passive dispersal ability of freshwater snails [70] and the frequent connection between the island and the continent. Such diversification within the island is caused by various mechanisms [2–4], but it is difficult to identify the mechanism based on our results. Nevertheless, some regional clades in our phylogeny suggest that geographical structure within the Japanese archipelago contributed to the diversification.

The time of the immigration and diversification estimated based our analyses is clearly more recent than that estimated by phylogeography of most freshwater fishes in Japan. Most divergence times within the Japanese archipelago of widely distributed strictly freshwater fish species are estimated to precede 1.7 Ma (e.g. [44, 47, 48, 50, 51, 53, 101]). As a consequence, the colonization from the continent occurred earlier. This difference between freshwater snails and fishes may be derived from differences in their ability and mode of dispersal. As noted above, freshwater snails have low active and high passive dispersal potential [70]. On the other hand, fish have strong active dispersal potential within well-connected river and wetland systems [102, 103], but have limited dispersal potential within vicariant water systems [104–106]. Additional taxon sampling and detailed analyses of population genetics are required to clarify the diversification mechanisms and biogeographic history of planorbids. However, our study shows that diversification occurred after the separation of the islands from the continent, an event that was crucial for creating the diversity of freshwater organisms in the Japanese archipelago today.

## Conclusions

Our results have shown that most of the planorbid lineages in the Japanese archipelago have closely related groups on the continent. In each lineage, different biogeographical patterns were detected via phylogenetic analysis. In addition, the branches between the Japanese archipelago populations and the continental populations date back to 1.7 Ma before the Japanese archipelago formed as an “archipelago.” On the other hand, our analysis showed that the present diversity of Japanese planorbid lineages is mainly the result of differentiation within the Japanese archipelago. Although additional taxon sampling and genetic analysis focused on each lineage are necessary, our study shows that diversification within the islands is more crucial to creating the present diversity than the diversity that existed when the islands were not separated from the continent.

## Additional files

Additional file 1: Sample information of Planorbidae in this study. See also Fig. 1. TUMC samples were deposited in the Tohoku University Museum Collection, Tohoku University; OKCABM samples were deposited in the Laboratory of Conservation of Aquatic Biodiversity, Faculity of Agriculture, Okayama University. (XLSX 26 kb)

Additional file 2: Aligned sequences of 16S after selecting by trimAl. (TXT 88 kb)

Additional file 3: The Bayesian phylogenetic tree inferred from mtocondorial CO1. Each number and colour at the terminal branch of the tree indicates the sample number, species name and collected region (Fig. 1 and Additional file 1). Numbers at the branch nodes represent BPP, MLBV, and NJ. On the right side, the vertical bars indicate nominal clades. (PDF 267 kb)

Additional file 4: The Bayesian phylogenetic tree inferred from 16S. Each number and colour at the terminal branch of the tree indicates the sample number, species name and collected region (Fig. 1 and Additional file 1). Numbers at the branch nodes represent BPP, MLBV, and NJ. On the right side, the vertical bars indicate nominal clades. (PDF 267 kb)

Additional file 5: The Bayesian phylogenetic tree inferred from H3. Each number and colour at the terminal branch of the tree indicates the sample number, species name and collected region (Fig. 1 and Additional file 1). Numbers at the branch nodes represent BPP, MLBV, and NJ. On the right side, the vertical bars indicate nominal clades. (PDF 263 kb)

Additional file 6: Detailed information of divergence time estimation, ancestral state reconstruction, and lineage diversity estimation. See also Table 4. (XLSX 56 kb)

## Abbreviations

16S: Large ribosomal subunit
BI: Bayesian inference
BPP: Bayesian posterior probabilities
BV: Bootstrap value
CO1: Cytochrome c oxidase subunit 1
ESS: Effective sample size
H3: Histone 3
Ma: Mega annum
ML: Maximum likelihood
NJ: Neighbour-joining
PCR: Polymerase chain reaction
