# Supplementary material for: Phylogeography of freshwater planorbid snails reveals diversification patterns in Eurasian continental islands
Source: BMC Evol Biol. 2018 Nov 6;18:164. doi: 10.1186/s12862-018-1273-3 (PMC6219199; doi:10.1186/s12862-018-1273-3)
Supplement: Supplementary file 3 — The Bayesian phylogenetic tree inferred from mtocondorial CO1. Each number and colour at the terminal branch of the tree indicates the sample number, species name and collected region (Fig. 1 and Additional file 1). Numbers at the branch nodes represent BPP, MLBV, and NJ. On the right side, the vertical bars indicate nominal clades. (PDF 267 kb) [file 12862_2018_1273_MOESM3_ESM.pdf]

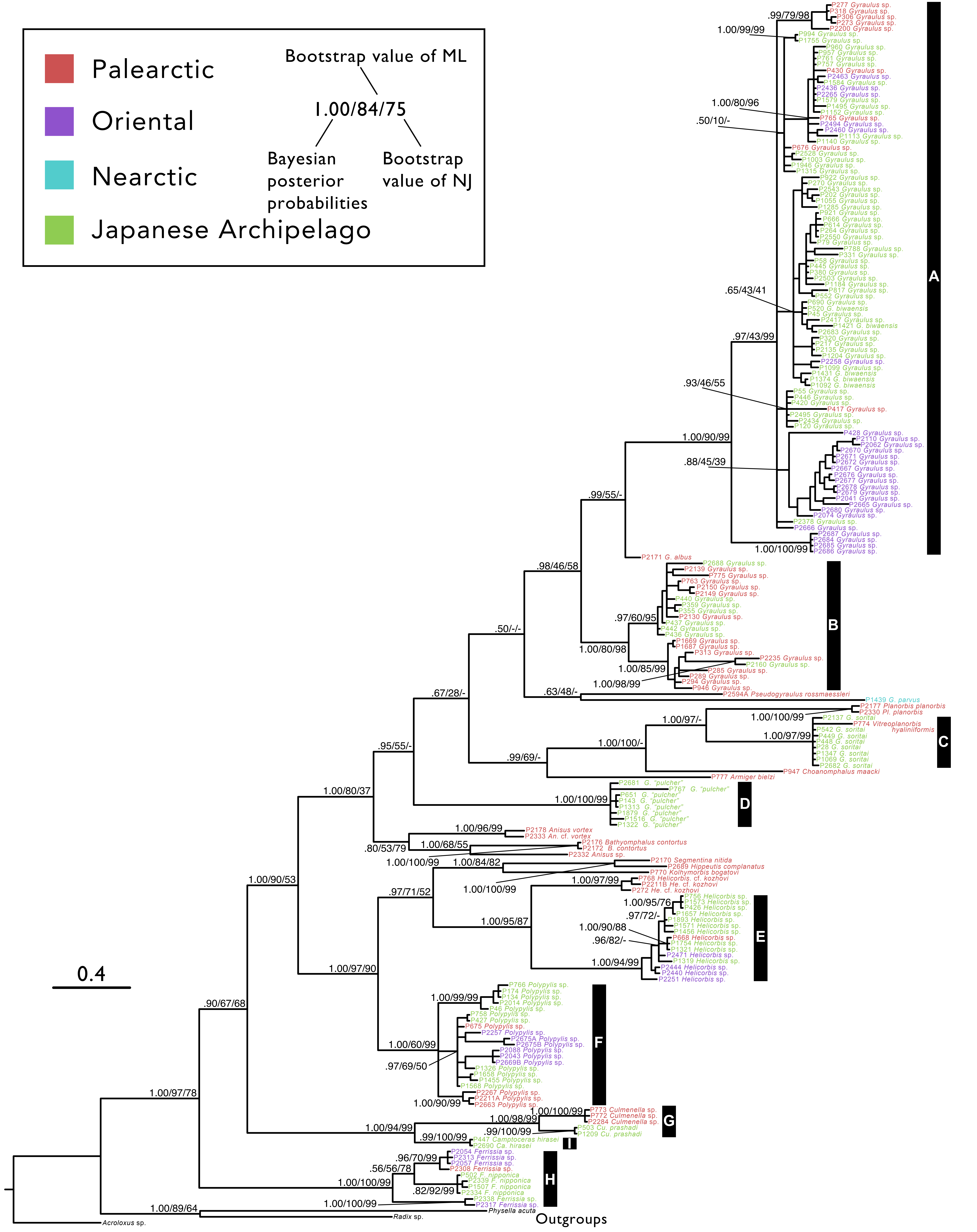

Palearctic
Oriental
Nearctic
Japanese Archipelago
Bootstrap value of ML
1.00/84/75
Bayesian posterior probabilities
Bootstrap value of NJ
0.4
A
B
C
D
E
F
G
H
I
Outgroups
P277 Gyraulus sp.
P318 Gyraulus sp.
P306 Gyraulus sp.
P273 Gyraulus sp.
P2200 Gyraulus sp.
P994 Gyraulus sp.
P1755 Gyraulus sp.
P960 Gyraulus sp.
P957 Gyraulus sp.
P761 Gyraulus sp.
P757 Gyraulus sp.
P430 Gyraulus sp.
P2463 Gyraulus sp.
P1584 Gyraulus sp.
P2436 Gyraulus sp.
P2265 Gyraulus sp.
P1579 Gyraulus sp.
P1495 Gyraulus sp.
P1152 Gyraulus sp.
P765 Gyraulus sp.
P2494 Gyraulus sp.
P2460 Gyraulus sp.
P1113 Gyraulus sp.
P1140 Gyraulus sp.
P676 Gyraulus sp.
P2528 Gyraulus sp.
P1003 Gyraulus sp.
P1946 Gyraulus sp.
P1315 Gyraulus sp.
P922 Gyraulus sp.
P270 Gyraulus sp.
P2543 Gyraulus sp.
P202 Gyraulus sp.
P1055 Gyraulus sp.
P1285 Gyraulus sp.
P921 Gyraulus sp.
P666 Gyraulus sp.
P614 Gyraulus sp.
P264 Gyraulus sp.
P2550 Gyraulus sp.
P79 Gyraulus sp.
P788 Gyraulus sp.
P331 Gyraulus sp.
P58 Gyraulus sp.
P445 Gyraulus sp.
P380 Gyraulus sp.
P2503 Gyraulus sp.
P1184 Gyraulus sp.
P817 Gyraulus sp.
P552 Gyraulus sp.
P690 Gyraulus sp.
P520 G. biwaensis
P45 Gyraulus sp.
P2417 Gyraulus sp.
P1421 G. biwaensis
P2683 Gyraulus sp.
P320 Gyraulus sp.
P217 Gyraulus sp.
P2135 Gyraulus sp.
P1204 Gyraulus sp.
P2258 Gyraulus sp.
P1099 Gyraulus sp.
P1431 G. biwaensis
P1374 G. biwaensis
P1092 G. biwaensis
P55 Gyraulus sp.
P446 Gyraulus sp.
P420 Gyraulus sp.
P417 Gyraulus sp.
P2495 Gyraulus sp.
P2434 Gyraulus sp.
P120 Gyraulus sp.
P428 Gyraulus sp.
P2110 Gyraulus sp.
P2062 Gyraulus sp.
P2670 Gyraulus sp.
P2671 Gyraulus sp.
P2672 Gyraulus sp.
P2667 Gyraulus sp.
P2676 Gyraulus sp.
P2677 Gyraulus sp.
P2678 Gyraulus sp.
P2679 Gyraulus sp.
P2041 Gyraulus sp.
P2665 Gyraulus sp.
P2680 Gyraulus sp.
P2074 Gyraulus sp.
P2378 Gyraulus sp.
P2666 Gyraulus sp.
P2687 Gyraulus sp.
P2684 Gyraulus sp.
P2685 Gyraulus sp.
P2686 Gyraulus sp.
P2171 G. albus
P2688 Gyraulus sp.
P2139 Gyraulus sp.
P775 Gyraulus sp.
P763 Gyraulus sp.
P2150 Gyraulus sp.
P2149 Gyraulus sp.
P440 Gyraulus sp.
P359 Gyraulus sp.
P355 Gyraulus sp.
P2130 Gyraulus sp.
P437 Gyraulus sp.
P442 Gyraulus sp.
P436 Gyraulus sp.
P1669 Gyraulus sp.
P1687 Gyraulus sp.
P313 Gyraulus sp.
P2235 Gyraulus sp.
P2160 Gyraulus sp.
P285 Gyraulus sp.
P289 Gyraulus sp.
P294 Gyraulus sp.
P946 Gyraulus sp.
P2594A Pseudogyraulus rossmaessleri
P1439 G. parvus
P2177 Planorbis planorbis
P2330 Pl. planorbis
P2137 G. soritai
P774 Vitreoplanorbis hyaliniiformis
P542 G. soritai
P449 G. soritai
P448 G. soritai
P28 G. soritai
P1347 G. soritai
P1069 G. soritai
P2682 G. soritai
P947 Choanomphalus maacki
P777 Armiger bielzi
P2681 G. "pulcher"
P767 G. "pulcher"
P651 G. "pulcher"
P143 G. "pulcher"
P1313 G. "pulcher"
P1879 G. "pulcher"
P1516 G. "pulcher"
P1322 G. "pulcher"
P2178 Anisus vortex
P2333 An. cf. vortex
P2176 Bathyomphalus contortus
P2172 B. contortus
P2332 Anisus sp.
P2170 Segmentina nitida
P2689 Hippeutis complanatus
P770 Kolhymorbis bogatovi
P768 Helicorbis. cf. kozhovi
P2211B He. cf. kozhovi
P272 He. cf. kozhovi
P756 Helicorbis sp.
P1573 Helicorbis sp.
P426 Helicorbis sp.
P1657 Helicorbis sp.
P1893 Helicorbis sp.
P1571 Helicorbis sp.
P1456 Helicorbis sp.
P668 Helicorbis sp.
P1754 Helicorbis sp.
P1321 Helicorbis sp.
P2471 Helicorbis sp.
P1319 Helicorbis sp.
P2444 Helicorbis sp.
P2440 Helicorbis sp.
P2251 Helicorbis sp.
P766 Polypylis sp.
P174 Polypylis sp.
P134 Polypylis sp.
P2014 Polypylis sp.
P46 Polypylis sp.
P758 Polypylis sp.
P427 Polypylis sp.
P675 Polypylis sp.
P2257 Polypylis sp.
P2675A Polypylis sp.
P2675B Polypylis sp.
P2088 Polypylis sp.
P2043 Polypylis sp.
P2669B Polypylis sp.
P1326 Polypylis sp.
P1658 Polypylis sp.
P1455 Polypylis sp.
P1568 Polypylis sp.
P2267 Polypylis sp.
P2211A Polypylis sp.
P2663 Polypylis sp.
P773 Culmenella sp.
P772 Culmenella sp.
P2284 Culmenella sp.
P503 Cu. prashadi
P1209 Cu. prashadi
P447 Camptoceras hirasei
P2690 Ca. hirasei
P2054 Ferrissia sp.
P2313 Ferrissia sp.
P2057 Ferrissia sp.
P2308 Ferrissia sp.
P502 F. nipponica
P2339 F. nipponica
P1507 F. nipponica
P2334 F. nipponica
P2338 Ferrissia sp.
P2317 Ferrissia sp.
Physella acuta
Radix sp.
Acroloxus sp.
.99/79/98
1.00/99/99
1.00/80/96
.50/10/-
.65/43/41
.97/43/99
.93/46/55
1.00/90/99
.88/45/39
.99/55/-
1.00/100/99
.98/46/58
.97/60/95
1.00/80/98
1.00/85/99
1.00/98/99
.50/-/-
.63/48/-
.67/28/-
1.00/100/99
1.00/97/-
1.00/97/99
1.00/100/-
.99/69/-
.95/55/-
1.00/80/37
1.00/100/99
1.00/96/99
1.00/68/55
.80/53/79
1.00/100/99
1.00/84/82
1.00/90/53
1.00/100/99
1.00/97/99
.97/71/52
1.00/95/76
.97/72/-
1.00/95/87
1.00/90/88
.96/82/-
1.00/94/99
1.00/97/90
1.00/99/99
1.00/60/99
.97/69/50
1.00/90/99
.90/67/68
1.00/100/99
1.00/98/99
.99/100/99
1.00/94/99
.99/100/99
1.00/97/78
.96/70/99
.56/56/78
1.00/100/99
.82/92/99
1.00/100/99
1.00/89/64
