# Supplementary figures and images for: Phylogeography of freshwater planorbid snails reveals diversification patterns in Eurasian continental islands
Source: BMC Evol Biol. 2018 Nov 6;18:164. doi: 10.1186/s12862-018-1273-3 (PMC6219199; doi:10.1186/s12862-018-1273-3)

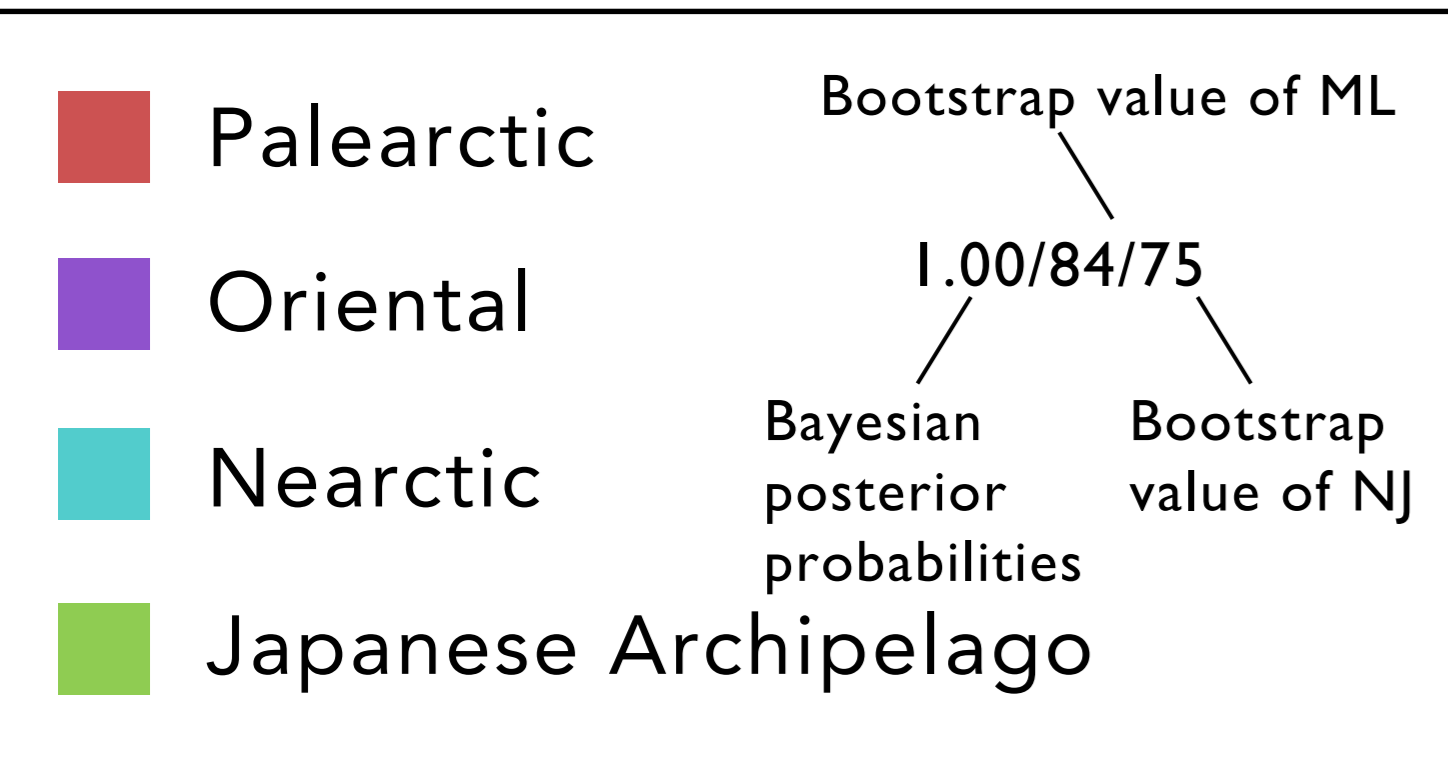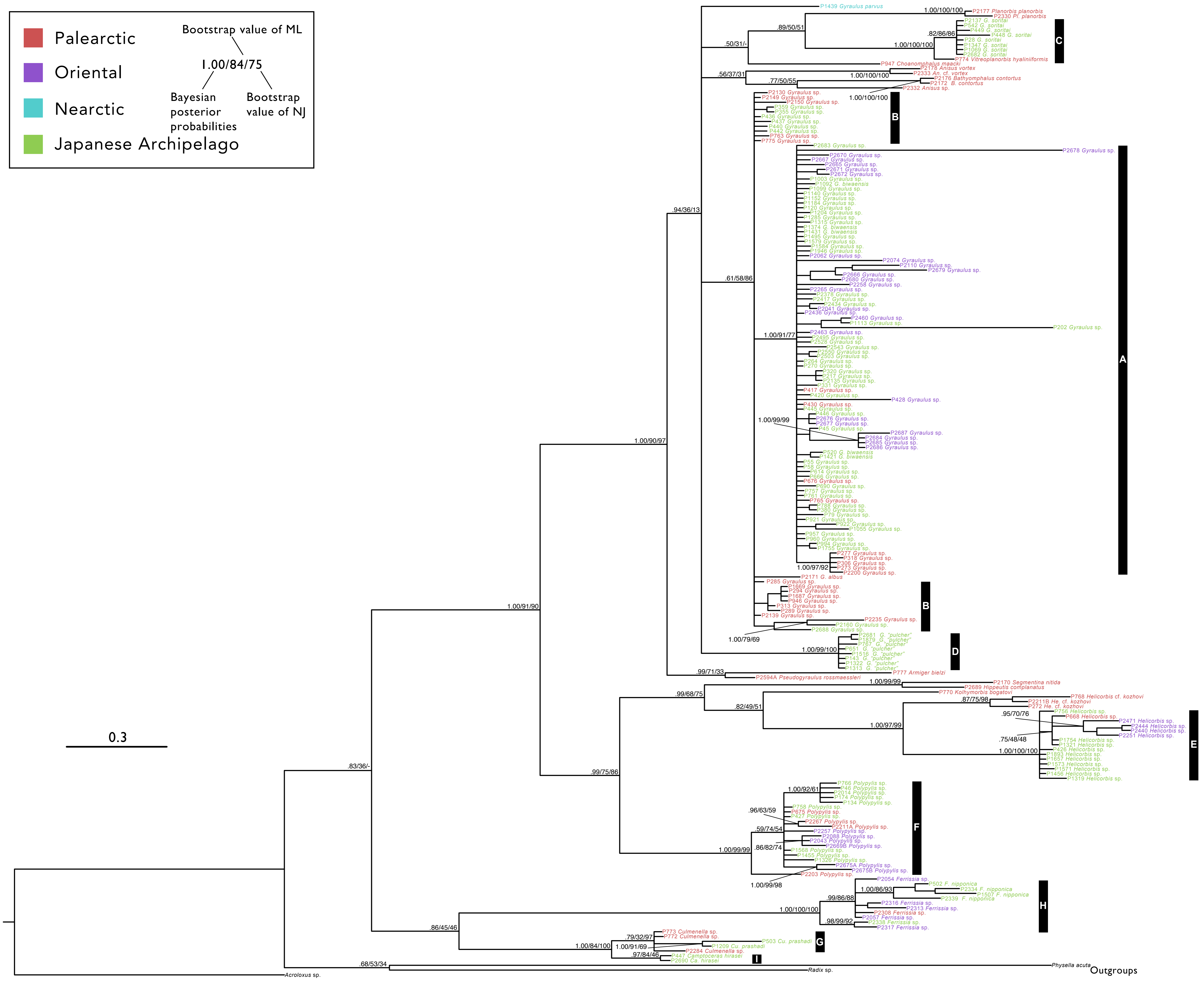

Supplement: Supplementary file 4 — The Bayesian phylogenetic tree inferred from 16S. Each number and colour at the terminal branch of the tree indicates the sample number, species name and collected region (Fig. 1 and Additional file 1). Numbers at the branch nodes represent BPP, MLBV, and NJ. On the right side, the vertical bars indicate nominal clades. (PDF 267 kb) [file 12862_2018_1273_MOESM4_ESM.pdf]

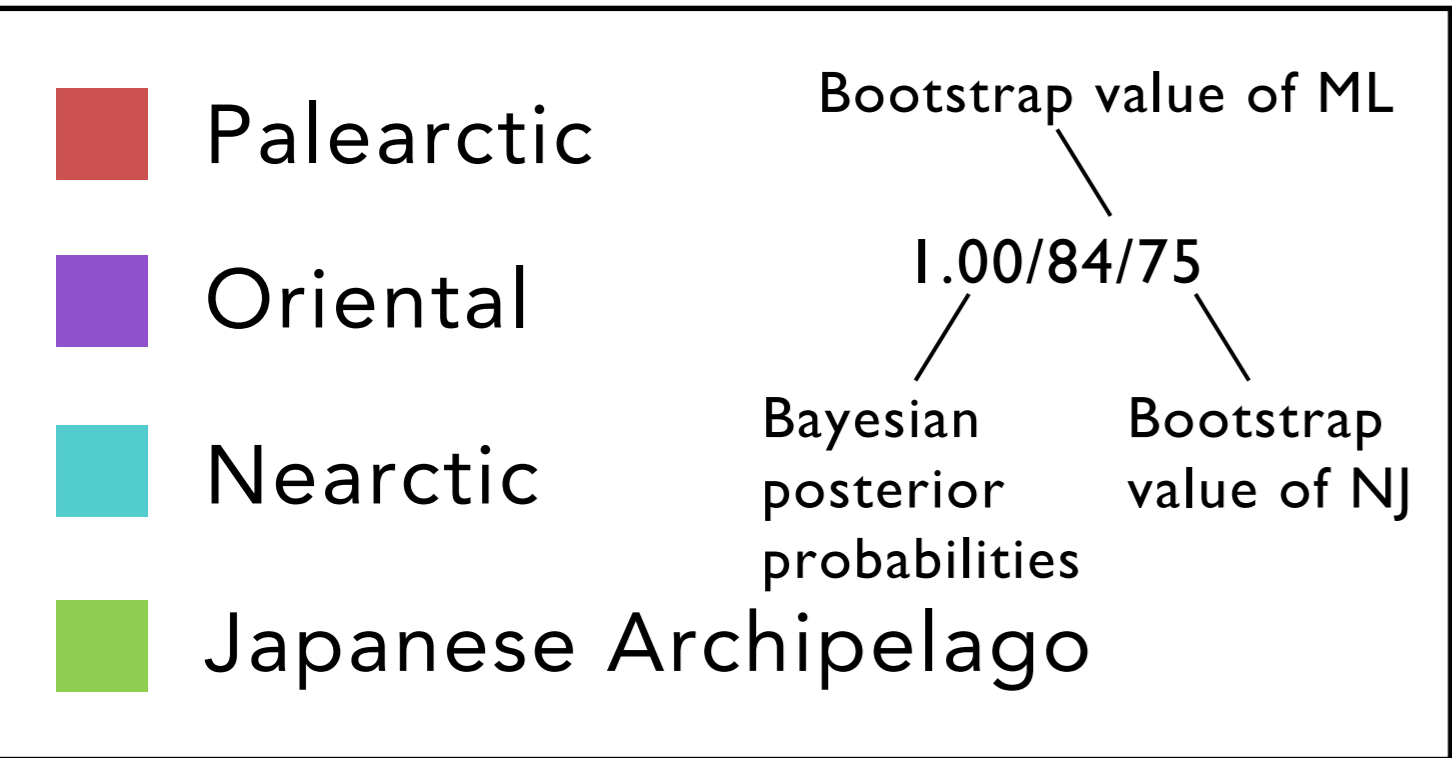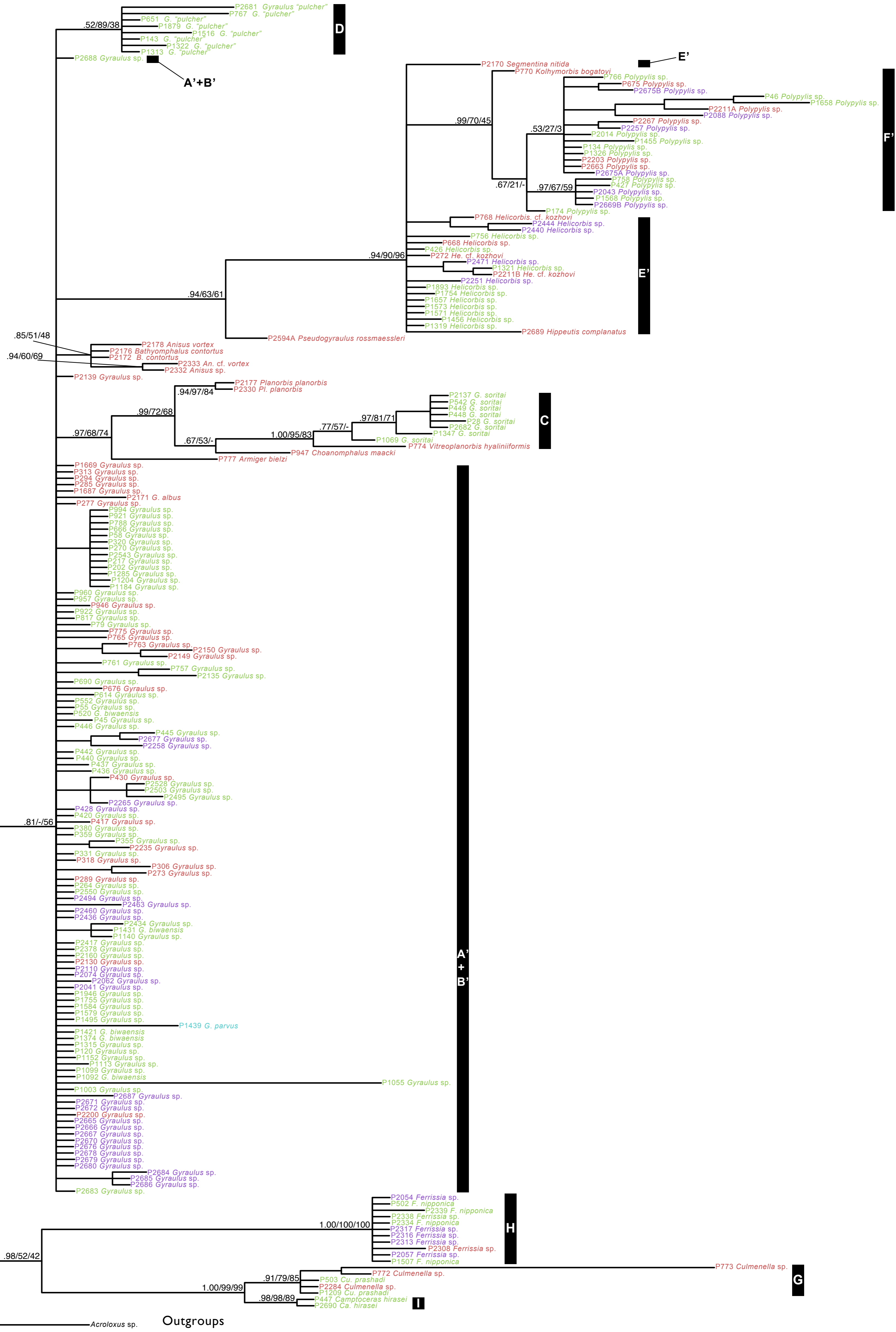

Supplement: Supplementary file 5 — The Bayesian phylogenetic tree inferred from H3. Each number and colour at the terminal branch of the tree indicates the sample number, species name and collected region (Fig. 1 and Additional file 1). Numbers at the branch nodes represent BPP, MLBV, and NJ. On the right side, the vertical bars indicate nominal clades. (PDF 263 kb) [file 12862_2018_1273_MOESM5_ESM.pdf]
